# Treatment Patterns and Survival Outcomes Among Patients With Hepatocellular Carcinoma

**DOI:** 10.1001/jamanetworkopen.2025.51665

**Published:** 2025-12-30

**Authors:** Kelsey S. Lau-Min, Angela C. Tramontano, Franklin Iheanacho, Thomas Adam Abrams, Christopher R. Manz

**Affiliations:** 1 Division of Hematology/Oncology, Department of Medicine, Massachusetts General Hospital, Boston; 2 Department of Medicine, Harvard Medical School, Boston, Massachusetts; 3 Department of Medical Oncology, Dana Farber Cancer Institute, Boston, Massachusetts; 4 Warren Alpert Medical School of Brown University, Providence, Rhode Island

## Abstract

**Importance:**

The treatment landscape for hepatocellular carcinoma (HCC) is rapidly evolving, with the approval of multiple systemic therapies for patients with advanced disease since 2017. However, ideal treatment sequencing approaches remain unknown, and data on clinical practice patterns are limited.

**Objective:**

To evaluate current treatment patterns, sequencing, and survival outcomes among patients receiving systemic therapy for HCC.

**Design, Setting, and Participants:**

This retrospective cohort study used data from a nationwide electronic health record (EHR)–derived database of more than 280 oncology practices at more than 800 US cancer clinics to assess adult patients who were treated with systemic therapy for HCC from January 1, 2011, to December 31, 2023.

**Exposure:**

First-line systemic therapy type.

**Main Outcomes and Measures:**

Overall survival (OS) was defined as the time from first-line systemic therapy initiation to death, last clinical activity, or the censor date of December 31, 2023. Progression-free survival (PFS) was defined as the time from first-line systemic therapy initiation to the earliest of EHR-documented disease progression, death, last clinical encounter, or the censor date.

**Results:**

Among 4198 patients (median [IQR] age, 67 [61-74] years; 3353 [79.9%] male), sorafenib was the most common first-line systemic therapy until it was overtaken by atezolizumab-bevacizumab in 2020; durvalumab-tremelimumab also became a common first-line therapy in 2023. Of the full study cohort, 871 (20.7%) received second-line therapy. Median OS was 8.1 months (95% CI, 7.7-8.6 months) but was not significantly associated with first-line systemic therapy type. Median PFS was 3.9 months (95% CI, 3.7-4.0 months); first-line atezolizumab-bevacizumab (hazard ratio, 0.84; 95% CI, 0.71-0.99) was associated with improved PFS compared with sorafenib.

**Conclusions and relevance:**

In this cohort study of patients treated with systemic therapy for HCC between 2011 and 2023, atezolizumab-bevacizumab and durvalumab-tremelimumab emerged as the most common first-line systemic therapies administered for HCC, but relatively few patients received second-line therapy. Additional research is needed to determine optimal treatment sequencing and increase the likelihood that patients may benefit from second-line systemic therapy options.

## Key Points

**Question:**

What are the current treatment patterns, sequencing, and survival outcomes among patients receiving systemic therapy for hepatocellular carcinoma (HCC)?

**Findings:**

In this cohort study of 4198 patients treated with systemic therapy for HCC between 2011 and 2023, atezolizumab-bevacizumab and durvalumab-tremelimumab replaced sorafenib as the most common first-line therapy over time; only 20% of patients received second-line therapy. Median overall survival was 8.1 months and was not significantly associated with first-line systemic therapy type.

**Meaning:**

These results suggest that the HCC treatment landscape has evolved considerably during the past decade, but more research is needed to determine optimal treatment sequencing.

## Teaser

This cohort study evaluates first-line systemic therapies and the need for second-line therapy in patients with hepatocellular carcinoma from 2011 through 2023.

## Introduction

Liver cancer is the sixth most frequently diagnosed cancer and the third most common cause of cancer-related death worldwide, with approximately 75% of cases attributable to hepatocellular carcinoma (HCC).^1,2^ Approximately 80% to 90% of patients with HCC have concomitant liver cirrhosis due to shared risk factors, such as heavy alcohol use, viral hepatitis, and metabolic dysfunction–associated steatotic liver disease.^3^ As a result, treatment for HCC must balance competing risks from both tumor progression and hepatic decompensation, with measures of liver function, such as the Child-Pugh score^4,5^ and albumin-bilirubin (ALBI) grade,^6^ routinely used to guide treatment decision-making due to their prognostic potential in patients with HCC.

Advanced HCC is characterized by portal vein invasion, extrahepatic spread, or intrahepatic disease that is not amenable to locoregional therapy (LRT), and survival for patients with advanced disease is often poor.^7^ Historically, the only systemic therapy option for patients with advanced HCC was sorafenib, which showed modest survival benefit compared with placebo and was approved in 2007.^8^ Starting in 2017, the US Food and Drug Administration (FDA) approved several new therapies for HCC.^9^ Only in more recent years were immunotherapy-based regimens, including atezolizumab-bevacizumab (in 2020) and durvalumab-tremelimumab (in 2022), approved after clinical trials demonstrated superior overall survival (OS) compared with sorafenib.^10,11^ As of early 2025, national best practice guidelines recommended atezolizumab-bevacizumab and durvalumab-tremelimumab as the preferred first-line systemic therapy options for patients with advanced HCC, although lenvatinib and sorafenib may be considered as alternatives for patients with contraindications to immunotherapy.^12^

As the HCC treatment landscape has evolved quickly, no randomized clinical trials have determined the ideal first-line treatment or optimal treatment sequencing after first-line therapy, and data on clinical practice patterns have been limited. We previously published treatment patterns for patients in the Surveillance, Epidemiology, and End Results (SEER)–Medicare database with a diagnosis of HCC between 2014 and 2019 and treatment claims through 2020, the most recent SEER-Medicare data as of February 2025.^13^ We found substantial changes in first-line systemic therapy patterns during the study period, with first-line sorafenib being replaced by a mix of newer therapies by 2020. However, the observation period ended 7 months after the approval of atezolizumab-bevacizumab and before the approval of durvalumab-tremelimumab, limiting insights into treatment patterns in the current era. To our knowledge, no other multi-institutional studies have evaluated treatment patterns in the current era after approval of both atezolizumab-bevacizumab and durvalumab-tremelimumab.

These data highlight the limited information available about current treatment patterns, sequencing, and outcomes for patients with HCC. Treatment patterns may offer important insights into the role of various systemic therapy regimens in the routine care of patients with advanced HCC. In addition, characterizing patient outcomes, such as OS and progression-free survival (PFS), may inform clinical decision-making where prospective clinical trial data are still lacking. Thus, we conducted a retrospective cohort study of a nationwide electronic health record (EHR)–derived oncology database to evaluate treatment patterns and survival outcomes among patients with HCC who were treated with systemic therapy between 2011 and 2023.

## Methods

### Data Source

This retrospective cohort study used data from the Flatiron Health Research Database, which includes deidentified EHR-derived data from more than 280 oncology practices at more than 800 cancer clinics throughout the US. The dataset includes patient-level structured and unstructured data that are curated using technology-enabled abstraction (eg, natural language processing) and strict quality control measures.^14,15,16,17,18,19^ This study was approved by the Dana-Farber Cancer Institute/Harvard Cancer Center institutional review board with a waiver of informed consent because there was no more than minimal risk to the research participants. The Strengthening the Reporting of Observational Studies in Epidemiology (STROBE) reporting guideline was used to guide research reporting.^20^

### Patient Population

We included adult patients with HCC who were treated with systemic therapy from January 1, 2011, to December 31, 2023. Patients were required to have an *International Classification of Diseases, Ninth Revision (ICD-9) *or* International Statistical Classification of Diseases and Related Health Problems, Tenth Revision (ICD-10) *code consistent with HCC, clinician-documented confirmation of HCC, and at least 2 clinic visits on different days to be included in the study cohort. The data cutoff date was December 31, 2023.

### HCC Treatment Patterns

We reviewed the Flatiron Health database for the following guideline-recommended regimens for HCC: anti–vascular endothelial growth factor or anti–vascular endothelial growth factor receptor therapy (ie, sorafenib, lenvatinib, cabozantinib, regorafenib, or ramucirumab), immunotherapy (ie, nivolumab, pembrolizumab, durvalumab-tremelimumab, or ipilimumab-nivolumab), and combination regimens (ie, atezolizumab-bevacizumab). We calculated the number (percentage) of patients who received each therapy in the first- and second-line settings. We plotted the distribution of first-line systemic therapy types over time and used the 3 most frequently administered first-line regimens (ie, atezolizumab-bevacizumab, lenvatinib, and sorafenib) to describe the most common first- to second-line treatment sequences. We also identified and calculated the number (percentage) of patients who received LRT before and after systemic therapy by modality (ie, ablation, stereotactic body radiotherapy, surgical resection, transarterial chemoembolization, transarterial radioembolization).

### Survival Outcomes

OS was defined as the time from first-line systemic therapy initiation to death, last clinical activity (including clinical encounters and laboratory or imaging visits), or the censor date of December 31, 2023; date of death was defined as a consensus variable derived from EHRs, US Social Security Death Index, and obituary data. PFS was defined as the time from first-line systemic therapy initiation to the earliest of EHR-documented disease progression, death, last clinical encounter, or the censor date.

### Potential Covariates

We evaluated multiple sociodemographic, clinical, and HCC-related factors as potential covariates in the association between first-line systemic therapy type and survival. Sociodemographic variables were EHR-derived and included age, sex, race (ie, Asian, Black or African American, White, other race [American Indian, Alaska Native, Native Hawaiian, Other Pacific Islander, or multiple race categories]), ethnicity, socioeconomic status quintile (calculated using the Yost Index derived from the US Census Bureau’s American Community Survey 2015-2019^21^), and insurance status. We collected sociodemographic data, including race and ethnicity, to assess for heterogeneity in treatment patterns and survival outcomes across these characteristics. Clinical characteristics included the Charlson Comorbidity Index (excluding cancer),^22^ Eastern Cooperative Oncology Group performance status, potential contributors to liver disease (ie, hepatitis B, hepatitis C, alcohol use, and obesity), Child-Pugh score (with a maximum of one additional point assigned for each of ascites and encephalopathy due to lack of data on severity),^4,5^ and documented history of ascites (as per clinician report or radiographic confirmation), encephalopathy (as per clinician report), and varices (as per *ICD-9* or *ICD-10* codes). HCC-specific characteristics included the year of advanced diagnosis, prior receipt of LRT, α-fetoprotein level, and ALBI grade.^6^ All characteristics were measured closest to and within 90 days before the date of first-line systemic therapy initiation except for the Charlson Comorbidity Index and history of varices, which were calculated using *ICD-9* and *ICD-10* codes documented within 1 year before first-line systemic therapy initiation.

### Statistical Analysis

We used descriptive statistics to characterize baseline sociodemographic, clinical, and HCC-related factors for the study cohort. We described patterns in the use of first- and second-line systemic therapy regimens and LRT over time. We used multivariable logistic generalized estimating equation models to evaluate the association between baseline characteristics at the start of first-line systemic therapy with subsequent receipt of second-line therapy, clustered by oncology practice. We then used Kaplan-Meier methods to estimate OS and PFS along with 95% CIs. We estimated the association between first-line systemic therapy type and OS and PFS, respectively, using separate multivariable Cox proportional hazards regression models, again clustered by oncology practice. Patients with missing laboratory data were excluded from the regression analyses. Models were adjusted for all clinically relevant covariates that have been shown in prior HCC studies^23,24^ to be associated with HCC and liver disease–related outcomes, except that ALBI was used in favor of the Childs-Pugh score due to a high degree of missingness. To address shorter follow-up time among patients who initiated systemic therapy in more recent years, we conducted sensitivity analyses that were limited to decedents to evaluate the robustness of our results. We used 2-sided tests of statistical significance and defined statistical significance as *P* < .05. All analyses were performed using SAS software, version 9.4 (SAS Institute Inc).

## Results

### Patient Population

The study included 4198 patients with HCC who were treated with systemic therapy (Table 1). The median (IQR) age was 67 (61-74) years. A total of 3353 patients (79.9%) were male, 844 (20.1%) were female, and 1 had unknown sex. As per EHR report, 220 (5.2%) were Asian, 496 (11.8%) were Black or African American, 2223 (53.0%) were White, 596 (14.2%) were of another race, and 663 (15.8%) were of unknown race. A total of 2732 patients (65.1%) were not of Hispanic or Latino ethnicity, and ethnicity was unknown for 995 patients (23.7%). A total of 3006 patients (71.6%) had a Charlson Comorbidity Index score of 0 to 1, and 3175 (75.6%) had a documented history of concomitant liver disease, with hepatitis C alone (967 [23.0%]) or hepatitis C and heavy alcohol use (851 [20.3%]) as the most common causes. Systemic therapy initiation year was relatively evenly distributed between 2011 and 2023, and 1602 patients (38.2%) had previously received LRT. The median (IQR) follow-up time was 7.9 (3.0-21.2) months.

**Table 1.** Baseline Characteristics of the Study Patients

Characteristic^a^	No. (%) (N = 4198)
Sociodemographic characteristics	
Age at first-line systemic therapy start date, y	
<50	129 (3.1)
50-64	1573 (37.5)
65-74	1565 (37.3)
≥75	931 (22.2)
Sex	
Female	844 (20.1)
Male	3353 (79.9)
Unknown	1 (0.0)
Race	
Asian	220 (5.2)
Black or African American	496 (11.8)
White	2223 (53.0)
Other race^b^	596 (14.2)
Unknown	663 (15.8)
Ethnicity	
Hispanic or Latino	471 (11.2)
Not Hispanic or Latino	2732 (65.1)
Unknown	995 (23.7)
Socioeconomic status	
1 (Lowest)	931 (22.2)
2	806 (19.2)
3	761 (18.1)
4	732 (17.4)
5 (Highest)	509 (12.1)
Unknown	459 (10.9)
Insurance status	
Commercial health plan	1225 (29.2)
Dual Medicare and Medicaid	215 (5.1)
Medicaid	302 (7.2)
Traditional Medicare	775 (18.5)
Medicare Advantage	695 (16.6)
Other	986 (23.5)
Clinical comorbidities	
Charlson Comorbidity Index	
0	1936 (46.1)
1	1070 (25.5)
≥2	1009 (24.0)
Unknown	183 (4.4)
Eastern Cooperative Oncology Group performance status	
0	989 (23.6)
1	1488 (35.4)
2	568 (13.5)
3	110 (2.6)
4	6 (0.1)
Unknown	1037 (24.7)
Concomitant liver disease	
Hepatitis B only	203 (4.8)
Hepatitis B and C	123 (2.9)
Hepatitis C only	967 (23.0)
Hepatitis C and heavy alcohol use	851 (20.3)
Heavy alcohol use	533 (12.7)
Heavy alcohol use and obesity	169 (4.0)
Obesity only	329 (7.8)
None	1023 (24.4)
Child-Pugh score	
A	1075 (25.6)
B	532 (12.7)
C	19 (0.5)
Unknown	2572 (61.3)
History of varices	210 (5.0)
History of ascites	1036 (24.7)
History of encephalopathy	231 (5.5)
Hepatocellular carcinoma characteristics	
Year of first-line therapy	
2011	107 (2.5)
2012	188 (4.5)
2013	257 (6.1)
2014	255 (6.1)
2015	301 (7.2)
2016	283 (6.7)
2017	337 (8.0)
2018	391 (9.3)
2019	369 (8.8)
2020	409 (9.7)
2021	425 (10.1)
2022	412 (9.8)
2023	464 (11.1)
Previously received locoregional therapy	1602 (38.2)
α-Fetoprotein, ng/mL	
<400	1864 (44.4)
≥400	1287 (30.7)
Unknown	1047 (24.9)
Albumin-bilirubin grade	
Grade 1 (≤−2.6 g/L)	1019 (24.3)
Grade 2 (−2.6 to −1.39 g/L)	2180 (51.9)
Grade 3 (>−1.39 g/L)	477 (11.4)
Unknown	522 (12.4)

^a^ See Methods section for definitions of all variables.

^b^ Other races included American Indian, Alaska Native, Native Hawaiian, other Pacific Islander, and multiple race categories.

### HCC Treatment Patterns

**Figure 1.**
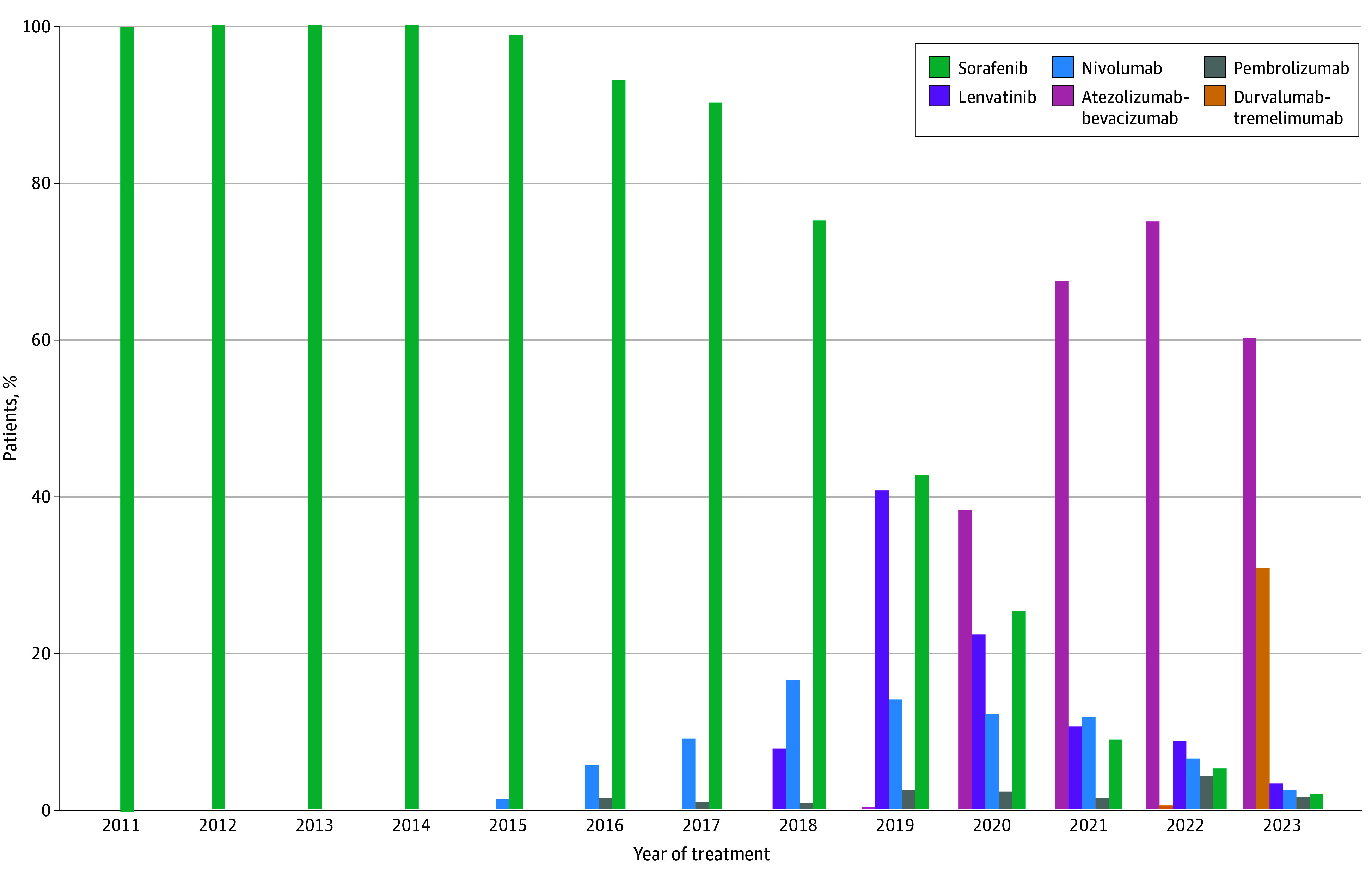
Patterns in First-Line Systemic Therapy Administration by Year

Sorafenib was the sole systemic therapy that was administered until 2015, when the immune checkpoint inhibitors nivolumab and pembrolizumab began to be administered (Figure 1; eTable 1 in Supplement 1). Lenvatinib emerged as the most frequently administered first-line systemic therapy in 2019 but was subsequently overtaken by atezolizumab-bevacizumab in 2020. Atezolizumab-bevacizumab has remained the predominant first-line systemic therapy since then and accounted for 60.0% of first-line systemic therapy administrations in 2023, although durvalumab-tremelimumab also emerged as a commonly administered first-line therapy in 2023 with 30.8% of first-line systemic therapy administrations in 2023. Among the 416 patients who initiated first-line atezolizumab-bevacizumab or durvalumab-tremelimumab in 2023, those who received atezolizumab-bevacizumab had better ALBI grade and were less likely to have commercial insurance than those who received durvalumab-tremelimumab (eTable 2 in Supplement 1). A total of 16 patients (5.8%) in the atezolizumab-bevacizumb group and 10 patients (7.1%) in the durvalumab-tremelimumab group had a history of varices (*P* = .61).

**Figure 2.**
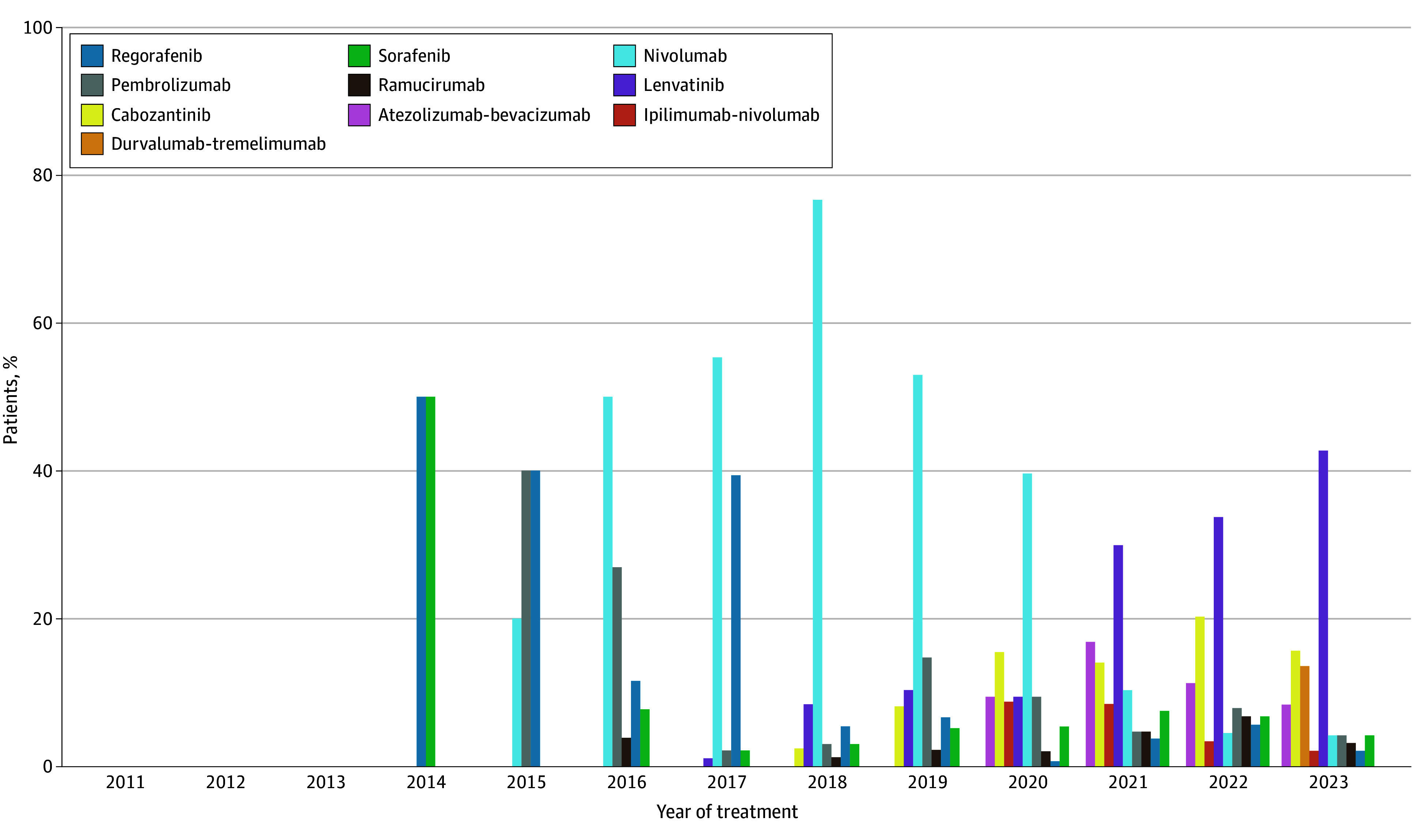
Patterns in Second-Line Systemic Therapy Administration by Year

Of the full study cohort, 871 (20.7%) received second-line systemic therapy; this number peaked at 173 of 389 patients (44.2%) who started first-line systemic therapy in 2018 but decreased thereafter (Figure 2; eTables 3 and 4 in Supplement 1). The predominant second-line therapy was nivolumab from 2016 to 2020 and then lenvatinib from 2021 to 2023. Lenvatinib was the most frequently administered second-line therapy regimen after progression on atezolizumab-bevacizumab, whereas there was greater heterogeneity in second-line therapy administered after lenvatinib or sorafenib (eFigure 1 and eTable 5 in Supplement 1). Patients who were in the highest socioeconomic status quintile, who had a history of concomitant hepatitis C and alcohol use (relative to no HCC risk factors), and who were treated in more recent years were more likely to receive second-line therapy, whereas patients with worse ALBI grade and history of ascites or encephalopathy were less likely to receive second-line systemic therapy (eTable 6 in Supplement 1).

A total of 683 patients (16.3%) received LRT after systemic therapy initiation during the study period, although this rate steadily decreased from 1019 (24.3%) in 2011 to 283 (6.7%) in 2023 (eFigure 2 and eTable 7 in Supplement 1). Transarterial therapies, such as transarterial chemoembolization and transarterial radioembolization, were the most frequently administered LRT modalities after systemic therapy initiation.

### Survival Outcomes

**Figure 3.**
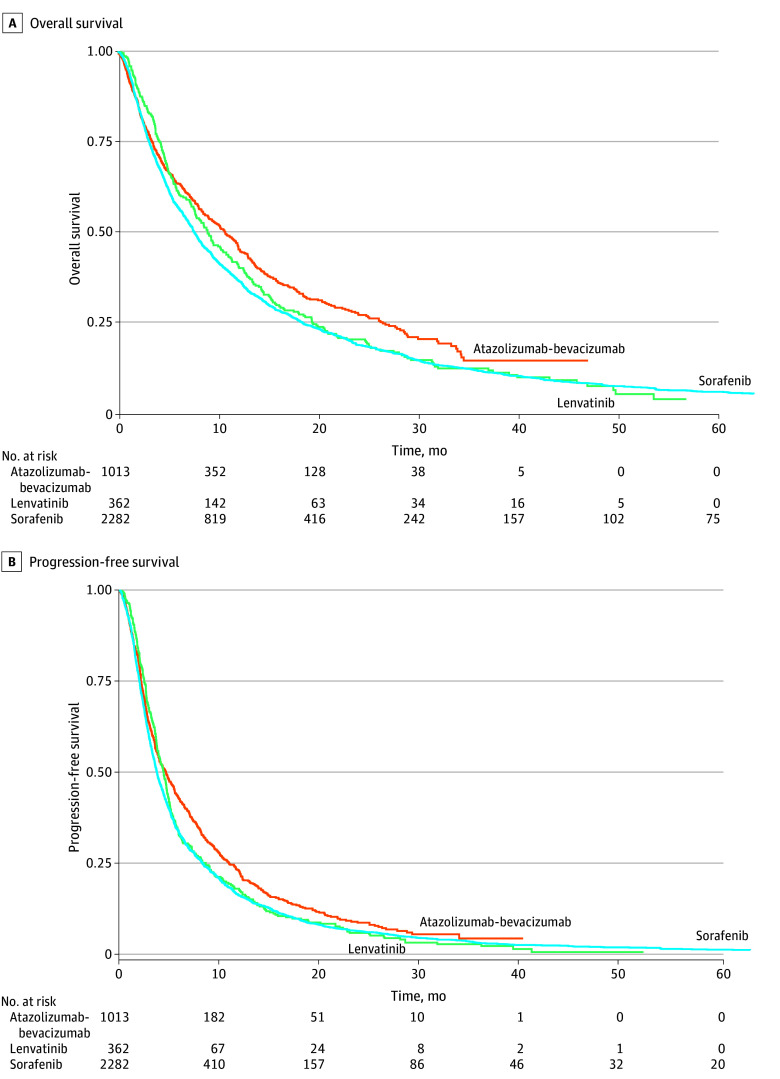
Overall and Progression-Free Survival After Initiating First-Line Systemic Therapy for Hepatocellular Carcinoma by First-Line Therapy Type

The median OS in the study cohort was 8.1 months (95% CI, 7.7-8.6 months). Although patients treated with first-line atezolizumab-bevacizumab qualitatively appeared to have higher OS compared with those treated with first-line lenvatinib and sorafenib (Figure 3A), there was no significant association between first-line systemic therapy type and OS after multivariable adjustment (Table 2; eTable 8 in Supplement 1). OS was, however, associated with a baseline Charlson Comorbidity Index score of 2 or higher (hazard ratio [HR], 1.15; 95% CI, 1.02-1.30; vs score 0) and ALBI grade (grade 2: HR, 1.69; 95% CI, 1.54-1.86; grade 3: HR, 2.65; 95% CI, 2.22-3.16; vs grade 1). The median PFS in the study cohort was 3.9 months (95% CI, 3.7-4.0 months). On multivariable analyses, first-line atezolizumab-bevacizumab (HR, 0.84; 95% CI, 0.71-0.99) was associated with improved PFS compared with sorafenib (Figure 3B, Table 2; eTable 9 in Supplement 1). Sensitivity analyses that were limited to decedents yielded similar results.

**Table 2.** Association Between First-Line Systemic Therapy Type and Overall and Progression-Free Survival^a^

Therapy	Overall survival		Progression-free survival	
	HR (95% CI)	*P* value	HR (95% CI)	*P* value
First-line therapy (reference, sorafenib)				
Atezolizumab-bevacizumab	0.93 (0.75-1.15)	.53	0.84 (0.70-0.99)	.04
Durvalumab-tremelimumab	1.09 (0.59-2.02)	.78	0.82 (0.53-1.26)	.36
Lenvatinib	1.03 (0.86-1.25)	.73	0.91 (0.76-1.09)	.32
Nivolumab	0.90 (0.76-1.08)	.26	0.82 (0.67-1.03)	.07
Pembrolizumab	0.99 (0.64-1.54)	.97	0.87 (0.60-1.27)	.47

Abbreviation: HR, hazard ratio.

^a^ Cox proportional hazards regression models were performed, adjusted for age, sex, race, ethnicity, socioeconomic status, insurance status, Charlson Comorbidity Index, concomitant liver disease, history of varices, ascites, or encephalopathy, year of advanced diagnosis, α-fetoprotein, and albumin-bilirubin grade. Patients with missing α-fetoprotein and albumin-bilirubin grade were excluded from these analyses.

## Discussion

In this study of patients with HCC treated with systemic therapy between 2011 and 2023, we observed substantial changes in treatment patterns during the study period. Atezolizumab-bevacizumab replaced sorafenib as the most common first-line therapy in 2020, with durvalumab-tremelimumab also becoming a common first-line therapy in 2023, whereas rates of LRT after systemic therapy initiation decreased substantially over time. Notably, most patients never received second-line systemic therapy despite the availability of multiple treatment options.

This study provides one of the most up-to-date overviews of the substantial changes in systemic therapy patterns for HCC during the last decade. The results are consistent with and build upon our prior work using the SEER-Medicare database of patients with HCC diagnosed through 2019 and treatment claims through 2020, which showed a mix of treatments starting to replace first-line sorafenib by the end of the study period.^13^ However, in the prior work,^13^ the observation period ended just 7 months after the approval of atezolizumab-bevacizumab in May 2020, whereas our current study demonstrates the emergence of atezolizumab-bevacizumab as the most common first-line therapy since that time. Durvalumab-tremelimumab received FDA approval in October 2022, was incorporated into national best practice guidelines shortly thereafter, and in our study accounted for 30.8% of first-line systemic therapy in 2023 compared with 60.0% for atezolizumab-bevacizumab. Clinical trials have demonstrated superior OS for both therapies compared with sorafenib, and both options are listed as preferred first-line systemic treatments in guidelines issued by the National Comprehensive Cancer Network.^10,11,12^ In this study, patients who received either option as first-line treatment in 2023 were similar across demographic and clinical characteristics, although histories of gastrointestinal bleeding and autoimmune disease were likely underreported in the EHR-derived database, thus underestimating these characteristics’ contribution to treatment preferences given that common contraindications to atezolizumab-bevacizumab and durvalumab-tremelimumab include untreated esophageal varices and uncontrolled autoimmune disease, respectively. Interestingly, rates of LRT administration after systemic therapy initiation have steadily decreased from 24.3% in 2011 to 6.7% in 2023, a trend that may be attributable to the availability of more effective systemic therapies for HCC over time.

Despite the rapid proliferation of new therapies for HCC between 2017 and 2022,^9^ our study demonstrated that only 1 in 5 patients who started systemic therapy received a second-line therapy, findings that are consistent with prior work.^13,25^ Receipt of second-line therapy peaked in the late 2010s, when sorafenib was still the preferred first-line therapy, perhaps due to rapid discontinuation of modestly effective sorafenib when newer second-line options emerged. However, second-line therapy administration has decreased in more recent years, which may be attributable to increased efficacy of first-line systemic therapies and/or limited follow-up time among more recently treated patients. Unsurprisingly, patients with indicators of worse liver function during first-line therapy were also less likely to receive second-line therapy in our study.

To our knowledge, there are no published randomized clinical trials and limited data on optimal treatment sequencing after first-line atezolizumab-bevacizumab and durvalumab-tremelimumab. The percentage of patients who received second-line therapy in our study was too low to provide meaningful insight into this important question. Lenvatinib was the most commonly used therapy after atezolizumab-bevacizumab, and there was insufficient patient volume and follow-up time to identify subsequent treatment after first-line durvalumab-tremelimumab. Patients who received lenvatinib or sorafenib most commonly received nivolumab or pembrolizumab monotherapy or atezolizumab-bevacizumab in the second line. This observation mostly reflects the transition years before the establishment of first-line atezolizumab-bevacizumab and durvalumab-tremelimumab as the standard of care because patients in 2021 to 2023 who received first-line lenvatinib or sorafenib would likely have had contraindications to immunotherapy. More research is needed to determine optimal treatment sequencing and strategies to increase the likelihood that patients benefit from second-line systemic therapy options.

In contrast to adjusted analyses in a prior SEER-Medicare study^13^ demonstrating that patients receiving atezolizumab-bevacizumab had superior OS to patients receiving sorafenib, the adjusted analyses in this study did not show superior OS of any therapy to sorafenib. Notably, this study was able to adjust for some clinical factors (eg, liver function via ALBI and tumor burden via α-fetoprotein levels) that are not available in claims-based studies, which may contribute to findings being discordant with SEER-Medicare analyses; it also includes patients of all ages and insurance types rather than being limited to older adults with Medicare. Our adjusted analyses, however, showed that atezolizumab-bevacizumab had superior PFS to sorafenib as first-line treatment, consistent with randomized clinical trial data demonstrating PFS benefit for atezolizumab-bevacizumab over sorafenib.^10^

### Limitations

This study has several limitations. It relies on an EHR-derived database from a cohort primarily composed of community oncology practices; the study population may not be representative of the US population of patients with HCC. Furthermore, the database may not capture care that occurs outside the EHR, particularly among patients who seek subsequent care at another institution, which may lead to underestimation of the amount of treatment received. However, compared with Medicare claims–based studies,^26,27^ it also includes adult patients of all ages and insurance types with more recent data to complement existing studies. As with other studies and data sources, the Flatiron Health database does not provide information on extent of disease, particularly in the liver, that is prognostically important in HCC and may influence survival patterns. However, we were able to adjust for laboratory values and some comorbidities, such as history of heavy alcohol use, that are often absent from other data sources. There was, however, a high degree of missingness for some clinical variables, which limited the robustness of our multivariable regression models.

## Conclusions

In this cohort study of patients treated with systemic therapy for HCC between 2011 and 2023, atezolizumab-bevacizumab and durvalumab-tremelimumab emerged as the most common first-line treatments for HCC. Many patients did not receive second-line treatment despite a multitude of available options. Further research, including the use of innovative approaches to combining clinically derived datasets and randomized clinical trials, is needed to determine optimal treatment sequencing as well as strategies to increase the likelihood that patients may benefit from second-line systemic therapy options.

## Notes

Supplement 1. **eTable 1.** Percentage of patients starting first-line systemic therapy administration, by year and treatment type **eTable 2.** Comparison of characteristics for patients starting first line atezolizumab/bevacizumab or durvalumab/tremelimumab in 2023 **eTable 3.** Percentage of patients starting each second-line systemic therapy, by year (%) **eTable 4.** Percentage of patients receiving second-line systemic therapy, by year of initiation of first-line therapy type **eTable 5.** Percentage of patients receiving second-line systemic therapy, by first-line therapy type **eTable 6.** Multivariable Logistic generalized estimating equation model output evaluating the association of characteristics at the start of first-line systemic therapy with receipt of second line therapy (n=2716) **eTable 7.** Percentage of patients who receive local therapy after first-line systemic therapy, by start year for first-line systemic therapy **eTable 8.** Multivariable Cox proportional hazards regression model outputs evaluating the association between first-line systemic therapy type and overall survival (n=2716) **eTable 9.** Multivariable Cox proportional hazards regression model outputs evaluating the association between first-line systemic therapy type and progression-free survival (n=2716) **eFigure 1.** Patterns in second-line systemic therapy administration, by first-line therapy type **eFigure 2.** Percentage of patients who received locoregional therapy after initiating systemic therapy for hepatocellular carcinoma.

Supplement 2.
Data Sharing Statement
