# Supplementary material for: Treatment Patterns and Survival Outcomes Among Patients With Hepatocellular Carcinoma
Source: JAMA Netw Open. 2025 Dec 30;8(12):e2551665. doi: 10.1001/jamanetworkopen.2025.51665 (PMC12754679; doi:10.1001/jamanetworkopen.2025.51665)
Supplement: Supplement 1. — eTable 1. Percentage of patients starting first-line systemic therapy administration, by year and treatment type eTable 2. Comparison of characteristics for patients starting first line atezolizumab/bevacizumab or durvalumab/tremelimumab in 2023 eTable 3. Percentage of patients starting each second-line systemic therapy, by year (%) eTable 4. Percentage of patients receiving second-line systemic therapy, by year of initiation of first-line therapy type eTable 5. Percentage of patients receiving second-line systemic therapy, by first-line therapy type eTable 6. Multivariable Logistic generalized estimating equation model output evaluating the association of characteristics at the start of first-line systemic therapy with receipt of second line therapy (n=2716) eTable 7. Percentage of patients who receive local therapy after first-line systemic therapy, by start year for first-line systemic therapy eTable 8. Multivariable Cox proportional hazards regression model outputs evaluating the association between first-line systemic therapy type and overall survival (n=2716) eTable 9. Multivariable Cox proportional hazards regression model outputs evaluating the association between first-line systemic therapy type and progression-free survival (n=2716) eFigure 1. Patterns in second-line systemic therapy administration, by first-line therapy type eFigure 2. Percentage of patients who received locoregional therapy after initiating systemic therapy for hepatocellular carcinoma. [file jamanetwopen-e2551665-s001.pdf]

## Supplemental Online Content

Lau-Min KS, Tramontano AC, Iheanacho F, Abrams TA, Manz CR. Treatment patterns and survival outcomes among patients with hepatocellular carcinoma. *JAMA Netw Open*. 2025;8(12):e2551665. doi:10.1001/jamanetworkopen.2025.51665

**eTable 1.** Percentage of patients starting first-line systemic therapy administration, by year and treatment type
**eTable 2.** Comparison of characteristics for patients starting first line atezolizumab/bevacizumab or durvalumab/tremelimumab in 2023
**eTable 3.** Percentage of patients starting each second-line systemic therapy, by year (%)
**eTable 4.** Percentage of patients receiving second-line systemic therapy, by year of initiation of first-line therapy type
**eTable 5.** Percentage of patients receiving second-line systemic therapy, by first-line therapy type
**eTable 6.** Multivariable Logistic generalized estimating equation model output evaluating the association of characteristics at the start of first-line systemic therapy with receipt of second line therapy (n=2716)
**eTable 7.** Percentage of patients who receive local therapy after first-line systemic therapy, by start year for first-line systemic therapy
**eTable 8.** Multivariable Cox proportional hazards regression model outputs evaluating the association between first-line systemic therapy type and overall survival (n=2716)
**eTable 9.** Multivariable Cox proportional hazards regression model outputs evaluating the association between first-line systemic therapy type and progression-free survival (n=2716)
**eFigure 1.** Patterns in second-line systemic therapy administration, by first-line therapy type
**eFigure 2.** Percentage of patients who received locoregional therapy after initiating systemic therapy for hepatocellular carcinoma

This supplemental material has been provided by the authors to give readers additional information about their work.

© 2025 Lau-Min KS et al. *JAMA Network Open*.

**eTable 1.** Percentage of patients starting first-line systemic therapy administration, by year and treatment type

| | 2011 | 2012 | 2013 | 2014 | 2015 | 2016 | 2017 | 2018 | 2019 | 2020 | 2021 | 2022 | 2023 |
|---|---|---|---|---|---|---|---|---|---|---|---|---|---|
| Sorafenib | 100 | 100 | 100 | 100 | 98.7 | 92.9 | 90 | 75.1 | 42.6 | 25.2 | 8.9 | 5.2 | 2 |
| Atezolizumab/bevacizumab | 0 | 0 | 0 | 0 | 0 | 0 | 0 | 0 | 0.3 | 38.1 | 67.4 | 74.9 | 60 |
| Durvalumab/tremelimumab | 0 | 0 | 0 | 0 | 0 | 0 | 0 | 0 | 0 | 0 | 0 | 0.5 | 30.8 |
| Lenvatinib | 0 | 0 | 0 | 0 | 0 | 0 | 0 | 7.7 | 40.7 | 22.3 | 10.6 | 8.7 | 3.3 |
| Nivolumab | 0 | 0 | 0 | 0 | 1.3 | 5.7 | 9 | 16.4 | 14.0 | 12.1 | 11.8 | 6.4 | 2.4 |
| Pembrolizumab | 0 | 0 | 0 | 0 | 0 | 1.4 | 0.9 | 0.8 | 2.5 | 2.2 | 1.4 | 4.2 | 1.5 |
| Total n | 107 | 188 | 257 | 255 | 301 | 282 | 333 | 389 | 364 | 404 | 417 | 403 | 458 |

© 2025 Lau-Min KS et al. *JAMA Network Open*.

**eTable 2.** Comparison of characteristics for patients starting first line atezolizumab/bevacizumab or durvalumab/tremelimumab in 2023

| | Total (N=416) | Atezolizumab, Bevacizumab (N=275) | Durvalumab, Tremelimumab (N=141) | P-value |
|---|---|---|---|---|
| Age, n (%) | | | | 0.93[1] |
| <50 years | 16 (3.8) | 10 (3.6) | 6 (4.3) | |
| 50-64 years | 116 (27.9) | 75 (27.3) | 41 (29.1) | |
| 65-74 years | 173 (41.6) | 114 (41.5) | 59 (41.8) | |
| >=75 years | 111 (26.7) | 76 (27.6) | 35 (24.8) | |
| Gender, n (%) | | | | 0.77[1] |
| Female | 91 (21.9) | 59 (21.5) | 32 (22.7) | |
| Male | 325 (78.1) | 216 (78.5) | 109 (77.3) | |
| Race, n (%) | | | | 0.72[1] |
| White | 233 (56.0) | 157 (57.1) | 76 (53.9) | |
| Black | 38 (9.1) | 27 (9.8) | 11 (7.8) | |
| Asian | 17 (4.1) | 11 (4.0) | 6 (4.3) | |
| Other Races/Unknown | 128 (30.8) | 80 (29.1) | 48 (34.0) | |
| Ethnicity, n (%) | | | | 0.81[1] |
| Hispanic | 48 (11.5) | 33 (12.0) | 15 (10.6) | |
| Non-Hispanic | 237 (57.0) | 158 (57.5) | 79 (56.0) | |
| Unknown | 131 (31.5) | 84 (30.5) | 47 (33.3) | |
| Insurance status, n (%) | | | | 0.005[1] |
| Commercial Health Plan | 102 (24.5) | 58 (21.1) | 44 (31.2) | |
| Dual Eligible | 25 (6.0) | 14 (5.1) | 11 (7.8) | |
| Medicaid | 21 (5.0) | 9 (3.3) | 12 (8.5) | |
| Medicare | 48 (11.5) | 32 (11.6) | 16 (11.3) | |
| Medicare Advantage | 105 (25.2) | 74 (26.9) | 31 (22.0) | |
| Other | 115 (27.6) | 88 (32.0) | 27 (19.1) | |
| SES Index, n (%) | | | | 0.57[1] |

© 2025 Lau-Min KS et al. *JAMA Network Open*.

| | Total (N=416) | Atezolizumab, Bevacizumab (N=275) | Durvalumab, Tremelimumab (N=141) | P-value |
|---|---|---|---|---|
| 1 - Lowest SES | 99 (23.8) | 59 (21.5) | 40 (28.4) | |
| 2 | 87 (20.9) | 58 (21.1) | 29 (20.6) | |
| 3 | 67 (16.1) | 49 (17.8) | 18 (12.8) | |
| 4 | 80 (19.2) | 52 (18.9) | 28 (19.9) | |
| 5 - Highest SES | 48 (11.5) | 32 (11.6) | 16 (11.3) | |
| Unknown | 35 (8.4) | 25 (9.1) | 10 (7.1) | |
| | | | | |
| Charlson Comorbidity, n (%) | | | | 0.84[1] |
| 0 | 175 (42.1) | 119 (43.3) | 56 (39.7) | |
| 1 | 112 (26.9) | 74 (26.9) | 38 (27.0) | |
| 2+ | 122 (29.3) | 77 (28.0) | 45 (31.9) | |
| Unknown | 7 (1.7) | 5 (1.8) | 2 (1.4) | |
| | | | | |
| Liver etiology history, n (%) | | | | 0.61[1] |
| None | 121 (29.1) | 82 (29.8) | 39 (27.7) | |
| Hepatitis B | 16 (3.8) | 13 (4.7) | 3 (2.1) | |
| Hepatitis B and C | 9 (2.2) | 5 (1.8) | 4 (2.8) | |
| Hepatitis C | 96 (23.1) | 64 (23.3) | 32 (22.7) | |
| Hep C and Alcohol Use | 66 (15.9) | 39 (14.2) | 27 (19.1) | |
| Heavy Alcohol Use | 54 (13.0) | 37 (13.5) | 17 (12.1) | |
| Alcohol Use and Obesity | 17 (4.1) | 9 (3.3) | 8 (5.7) | |
| Obesity | 37 (8.9) | 26 (9.5) | 11 (7.8) | |
| | | | | |
| Presence of ascites prior to systemic therapy, n (%) | | | | 0.07[1] |
| No/unknown | 324 (77.9) | 222 (80.7) | 102 (72.3) | |
| Yes | 70 (16.8) | 38 (13.8) | 32 (22.7) | |
| Missing | 22 (5.3) | 15 (5.5) | 7 (5.0) | |
| | | | | |
| Presence of encephalopathy prior to systemic therapy, n (%) | | | | 0.12[1] |
| No/unknown | 374 (89.9) | 251 (91.3) | 123 (87.2) | |

© 2025 Lau-Min KS et al. *JAMA Network Open*.

| | Total (N=416) | Atezolizumab, Bevacizumab (N=275) | Durvalumab, Tremelimumab (N=141) | P-value |
|---|---|---|---|---|
| Yes | 20 (4.8) | 9 (3.3) | 11 (7.8) | |
| Missing | 22 (5.3) | 15 (5.5) | 7 (5.0) | |
| | | | | |
| GI Bleed, n (%) | | | | 0.07[1] |
| No | 404 (97.1) | 270 (98.2) | 134 (95.0) | |
| Yes | 12 (2.9) | 5 (1.8) | 7 (5.0) | |
| | | | | |
| GI Varices, n (%) | | | | 0.61[1] |
| No | 390 (93.8) | 259 (94.2) | 131 (92.9) | |
| Yes | 26 (6.3) | 16 (5.8) | 10 (7.1) | |
| | | | | |
| Autoimmune disease, n (%) | | | | 0.56[1] |
| No | 404 (97.1) | 268 (97.5) | 136 (96.5) | |
| Yes | 12 (2.9) | 7 (2.5) | 5 (3.5) | |
| | | | | |
| Year of advanced HCC diagnosis, n (%) | | | | 0.83[1] |
| 2014 | 1 (0.2) | 1 (0.4) | 0 (0.0) | |
| 2015 | 1 (0.2) | 1 (0.4) | 0 (0.0) | |
| 2016 | 1 (0.2) | 1 (0.4) | 0 (0.0) | |
| 2017 | 2 (0.5) | 2 (0.7) | 0 (0.0) | |
| 2018 | 3 (0.7) | 2 (0.7) | 1 (0.7) | |
| 2019 | 3 (0.7) | 3 (1.1) | 0 (0.0) | |
| 2020 | 8 (1.9) | 5 (1.8) | 3 (2.1) | |
| 2021 | 19 (4.6) | 13 (4.7) | 6 (4.3) | |
| 2022 | 54 (13.0) | 38 (13.8) | 16 (11.3) | |
| 2023 | 324 (77.9) | 209 (76.0) | 115 (81.6) | |
| | | | | |
| Received Local Therapy Prior to Systemic Therapy, n (%) | | | | 0.12[1] |
| No | 280 (67.3) | 178 (64.7) | 102 (72.3) | |
| Yes | 136 (32.7) | 97 (35.3) | 39 (27.7) | |

© 2025 Lau-Min KS et al. *JAMA Network Open*.

| | Total (N=416) | Atezolizumab, Bevacizumab (N=275) | Durvalumab, Tremelimumab (N=141) | P-value |
|---|---|---|---|---|
| AFP, n (%) | | | | 0.33[1] |
| <400 | 165 (39.7) | 108 (39.3) | 57 (40.4) | |
| >=400 | 139 (33.4) | 87 (31.6) | 52 (36.9) | |
| Unknown | 112 (26.9) | 80 (29.1) | 32 (22.7) | |
| ALBI Grade, n (%) | | | | 0.006[1] |
| Grade 1 (<= -2.6) | 138 (33.2) | 104 (37.8) | 34 (24.1) | |
| Grade 2 (-2.6 to -1.39) | 224 (53.8) | 137 (49.8) | 87 (61.7) | |
| Grade 3 (> -1.39) | 25 (6.0) | 12 (4.4) | 13 (9.2) | |
| Unknown | 29 (7.0) | 22 (8.0) | 7 (5.0) | |
| Baseline ECOG, n (%) | | | | 0.69[1] |
| 0 | 97 (23.3) | 63 (22.9) | 34 (24.1) | |
| 1 | 139 (33.4) | 87 (31.6) | 52 (36.9) | |
| 2 | 61 (14.7) | 40 (14.5) | 21 (14.9) | |
| 3+ | 8 (1.9) | 5 (1.8) | 3 (2.1) | |
| Missing | 2 (0.5) | 1 (0.4) | 1 (0.7) | |
| Child-Pugh score, Median (IQR) | 6.0 (1.0) | 5.0 (1.0) | 6.0 (2.0) | 0.005[2] |
| Child-Pugh score, n (%) | | | | 0.0005[1] |
| A | 118 (28.4) | 76 (27.6) | 42 (29.8) | |
| B | 31 (7.5) | 11 (4.0) | 20 (14.2) | |
| Missing | 267 (64.2) | 188 (68.4) | 79 (56.0) | |

[1]Chi-Square p-value; [2]Kruskal-Wallis p-value;

© 2025 Lau-Min KS et al. *JAMA Network Open*.

**eTable 3.** Percentage of patients starting each second-line systemic therapy, by year (%)

| | 2011 | 2012 | 2013 | 2014 | 2015 | 2016 | 2017 | 2018 | 2019 | 2020 | 2021 | 2022 | 2023 |
|---|---|---|---|---|---|---|---|---|---|---|---|---|---|
| Sorafenib | | | | 50 | 0 | 7.7 | 2.1 | 3.0 | 5.2 | 5.4 | 7.5 | 6.7 | 4.2 |
| Atezolizumab/bevacizumab | | | | 0 | 0 | 0 | 0 | 0 | 0 | 9.4 | 16.8 | 11.2 | 8.3 |
| Cabozantinib | | | | 0 | 0 | 0 | 0 | 2.4 | 8.1 | 15.4 | 14.0 | 20.2 | 15.6 |
| Durvalumab/tremelimumab | | | | 0 | 0 | 0 | 0 | 0 | 0 | 0 | 0 | 0 | 13.5 |
| Ipilimumab/nivolumab | | | | 0 | 0 | 0 | 0 | 0 | 0 | 8.7 | 8.4 | 3.4 | 2.1 |
| Lenvatinib | | | | 0 | 0 | 0 | 1.1 | 8.4 | 10.3 | 9.4 | 29.9 | 33.7 | 42.7 |
| Nivolumab | | | | 0 | 20 | 50 | 55.3 | 76.7 | 52.9 | 39.6 | 10.3 | 4.5 | 4.2 |
| Pembrolizumab | | | | 0 | 40 | 26.9 | 2.1 | 3 | 14.7 | 9.4 | 4.7 | 7.9 | 4.2 |
| Ramucirumab | | | | 0 | 0 | 3.9 | 0 | 1.2 | 2.2 | 2.0 | 4.7 | 6.7 | 3.1 |
| Regorafenib | | | | 50 | 40 | 11.5 | 39.4 | 5.5 | 6.6 | 0.7 | 3.7 | 5.6 | 2.1 |
| Total n | -- | -- | -- | 2 | 5 | 26 | 94 | 167 | 136 | 149 | 107 | 89 | 96 |

© 2025 Lau-Min KS et al. *JAMA Network Open*.

**eTable 4.** Percentage of patients receiving second-line systemic therapy, by year of initiation of first-line therapy type

| Year of First-line therapy | Received second-line therapy (N (%) |
|---|---|
| 2011 | 0 (0) |
| 2012 | 3 (1.6) |
| 2013 | 7 (2.7) |
| 2014 | 8 (3.1) |
| 2015 | 29 (9.6) |
| 2016 | 56 (19.8) |
| 2017 | 116 (34.4) |
| 2018 | 173 (44.2) |
| 2019 | 154 (41.7) |
| 2020 | 118 (28.9) |
| 2021 | 99 (23.3) |
| 2022 | 74 (18.0) |
| 2023 | 34 (7.3) |
| Total | 871 |

© 2025 Lau-Min KS et al. *JAMA Network Open*.

**eTable 5.** Percentage of patients receiving second-line systemic therapy, by first-line therapy type

| | First-line therapy | | |
|---|---|---|---|
| Second-line therapy | Atezolizumab/bevacizumab | Lenvatinib | Sorafenib |
| None | 83.0 | 67.9 | 61.5 |
| Atezolizumab/bevacizumab | 0 | 8.2 | 8.9 |
| Lenvatinib | 8.9 | 0 | 4.1 |
| Sorafenib | 1.7 | 2.2 | 0 |
| Cabozantinib | 2.8 | 7.6 | 3.0 |
| Nivolumab or Pembrolizumab | 0.6 | 8.7 | 16.6 |
| Ipilimumab/nivolumab | 0.5 | 3.3 | 4.1 |
| Other | 2.5 | 2.1 | 1.8 |

© 2025 Lau-Min KS et al. *JAMA Network Open*.

**eTable 6.** Multivariable Logistic generalized estimating equation model output evaluating the association of characteristics at the start of first-line systemic therapy with receipt of second line therapy (n=2716)

| | OR | 95% CI | | p-value |
|---|---|---|---|---|
| | | Lower limit | Upper limit | |
| First-line therapy (ref=Sorafenib) | | | | |
| Atezolizumab/bevacizumab | 0.33 | 0.25 | 0.43 | <.0001 |
| Durvalumab/tremelimumab | 0.04 | 0.01 | 0.15 | <.0001 |
| Lenvatinib | 0.79 | 0.62 | 1.01 | 0.06 |
| Nivolumab | 0.33 | 0.24 | 0.45 | <.0001 |
| Pembrolizumab | 0.24 | 0.09 | 0.65 | 0.005 |
| Age (ref=<50) | | | | |
| 50-64 | 1.30 | 0.73 | 2.32 | 0.37 |
| 65-74 | 1.05 | 0.59 | 1.89 | 0.87 |
| >=75 | 0.98 | 0.52 | 1.85 | 0.96 |
| Female (ref=Male) | 1.27 | 0.99 | 1.62 | 0.06 |
| Race (ref=White) | | | | |
| Asian | 1.44 | 0.96 | 2.16 | 0.08 |
| Black | 1.08 | 0.76 | 1.55 | 0.66 |
| Other race/unknown | 0.96 | 0.70 | 1.30 | 0.77 |
| Ethnicity (ref=Not Hispanic) | | | | |
| Hispanic | 1.18 | 0.84 | 1.67 | 0.34 |
| Unknown | 0.89 | 0.63 | 1.25 | 0.49 |
| Charlson comorbidity index (ref=0) | | | | |
| 1 | 0.89 | 0.71 | 1.12 | 0.31 |
| 2+ | 0.89 | 0.69 | 1.15 | 0.36 |
| Unknown | 0.58 | 0.33 | 1.01 | 0.05 |
| Insurance (ref=Commercial) | | | | |
| Dual Medicare and Medicaid | 1.13 | 0.68 | 1.88 | 0.63 |
| Medicaid | 0.98 | 0.70 | 1.37 | 0.90 |
| Traditional Medicare | 1.30 | 0.92 | 1.82 | 0.13 |
| Medicare Advantage | 1.18 | 0.81 | 1.70 | 0.39 |
| Other | 1.04 | 0.76 | 1.44 | 0.80 |
| SES (ref=1-Lowest) | | | | |
| 2 | 0.94 | 0.66 | 1.33 | 0.71 |
| 3 | 1.20 | 0.81 | 1.78 | 0.36 |
| 4 | 1.06 | 0.79 | 1.44 | 0.68 |
| 5 - Highest SES | 1.45 | 1.04 | 2.02 | 0.03 |
| Unknown | 0.91 | 0.56 | 1.50 | 0.72 |
| Liver history (ref=None) | | | | |
| Hepatitis B only | 1.25 | 0.74 | 2.11 | 0.40 |
| Hepatitis B and C | 1.26 | 0.69 | 2.28 | 0.45 |
| Hepatitis C only | 1.05 | 0.78 | 1.41 | 0.77 |
| Hepatitis C and heavy alcohol use | 1.51 | 1.13 | 2.01 | 0.005 |
| Heavy alcohol use | 1.16 | 0.77 | 1.74 | 0.47 |
| Heavy alcohol use and obesity | 1.42 | 0.87 | 2.31 | 0.16 |
| Obesity only | 1.35 | 0.97 | 1.88 | 0.08 |
| First line therapy year (ref=2011-2016) | | | | |
| 2017-2020 | 10.76 | 7.81 | 14.83 | <.0001 |

© 2025 Lau-Min KS et al. *JAMA Network Open*.

| | | | | |
|---|---|---|---|---|
| 2020-2023 | 6.27 | 4.17 | 9.43 | <.0001 |
| ALBI grade (ref=1) | | | | |
| 2 | 0.59 | 0.45 | 0.78 | 0.002 |
| 3 | 0.31 | 0.22 | 0.46 | <.0001 |
| AFP (ref= <400) | | | | |
| >=400 | 1.08 | 0.89 | 1.30 | 0.45 |
| History of ascites (ref=No) | 0.59 | 0.44 | 0.80 | 0.006 |
| History of encephalopathy (ref=No) | 0.56 | 0.34 | 0.92 | 0.02 |
| History of varices (ref=No) | 1.35 | 0.93 | 1.94 | 0.11 |

OR = Odds Ratio; CI = Confidence Interval.
Patients with missing AFP and ALBI grade were excluded from these analyses. Years were combined due to low numbers in early years of the cohort causing the model to fail.

© 2025 Lau-Min KS et al. *JAMA Network Open*.

**eTable 7.** Percentage of patients who receive local therapy after first-line systemic therapy, by start year for first-line systemic therapy

| Local therapy | 2011 | 2012 | 2013 | 2014 | 2015 | 2016 | 2017 | 2018 | 2019 | 2020 | 2021 | 2022 | 2023 |
|---|---|---|---|---|---|---|---|---|---|---|---|---|---|
| All | 24.3 | 16.5 | 26.5 | 24.3 | 20.6 | 21.6 | 18.4 | 16.1 | 14.9 | 16.4 | 12.9 | 9.7 | 6.7 |
| TACE | 15.0 | 12.8 | 16.3 | 15.3 | 13.3 | 13.1 | 8.6 | 8.4 | 6.5 | 7.8 | 4.2 | 2.4 | 2.4 |
| TARE | 5.6 | 3.2 | 10.5 | 7.1 | 7.3 | 9.2 | 8.3 | 7.7 | 7.0 | 6.6 | 8.0 | 5.1 | 3.0 |
| Resection | 0.9 | 1.6 | 0 | 0.8 | 0.3 | 1.1 | 0.9 | 1.0 | 0 | 1.5 | 0.9 | 0.2 | 0 |
| SBRT | 1.9 | 0.5 | 3.5 | 4.3 | 3.3 | 3.2 | 2.4 | 1.3 | 2.7 | 3.2 | 1.2 | 3.2 | 1.3 |
| Ablation | 3.7 | 1.6 | 1.2 | 2.4 | 0.7 | 2.1 | 0.3 | 0.5 | 0.3 | 0 | 0.5 | 0 | 0 |

TACE = transarterial chemoembolization; TARE: transarterial radioembolization; SBRT = stereotactic body radiotherapy.

Column percentages may not add up to match Figure 4 column percentages because some patients received more than one local therapy at first-line systemic therapy.

© 2025 Lau-Min KS et al. *JAMA Network Open*.

**eTable 8.** Multivariable Cox proportional hazards regression model outputs evaluating the association between first-line systemic therapy type and overall survival (n=2716)

| | HR | 95% CI | | p-value |
|---|---|---|---|---|
| | | Lower limit | Upper limit | |
| First-line therapy (ref=Sorafenib) | | | | |
| Atezolizumab/bevacizumab | 0.93 | 0.76 | 1.15 | 0.53 |
| Durvalumab/tremelimumab | 1.09 | 0.59 | 2.02 | 0.78 |
| Lenvatinib | 1.03 | 0.86 | 1.25 | 0.73 |
| Nivolumab | 0.90 | 0.76 | 1.08 | 0.26 |
| Pembrolizumab | 0.99 | 0.64 | 1.54 | 0.97 |
| Age (ref=<50) | | | | |
| 50-64 | 0.91 | 0.69 | 1.19 | 0.48 |
| 65-74 | 0.92 | 0.72 | 1.19 | 0.53 |
| >=75 | 1.03 | 0.79 | 1.35 | 0.83 |
| Female (ref=Male) | 0.90 | 0.80 | 1.00 | 0.06 |
| Race (ref=White) | | | | |
| Asian | 0.85 | 0.68 | 1.06 | 0.16 |
| Black | 1.01 | 0.84 | 1.22 | 0.89 |
| Other race/unknown | 0.90 | 0.78 | 1.04 | 0.16 |
| Ethnicity (ref=Not Hispanic) | | | | |
| Hispanic | 0.86 | 0.72 | 1.02 | 0.08 |
| Unknown | 1.29 | 1.13 | 1.46 | 0.0001 |
| Charlson comorbidity index (ref=0) | | | | |
| 1 | 1.06 | 0.96 | 1.17 | 0.25 |
| 2+ | 1.15 | 1.02 | 1.30 | 0.02 |
| Unknown | 0.85 | 0.66 | 1.08 | 0.18 |
| Insurance (ref=Commercial) | | | | |
| Dual Medicare and Medicaid | 0.78 | 0.64 | 0.94 | 0.01 |
| Medicaid | 1.00 | 0.84 | 1.18 | 0.96 |
| Traditional Medicare | 0.96 | 0.81 | 1.13 | 0.60 |
| Medicare Advantage | 0.92 | 0.80 | 1.05 | 0.20 |
| Other | 1.01 | 0.88 | 1.16 | 0.94 |
| SES (ref=1-Lowest) | | | | |
| 2 | 1.05 | 0.89 | 1.24 | 0.54 |
| 3 | 1.02 | 0.90 | 1.15 | 0.79 |

© 2025 Lau-Min KS et al. *JAMA Network Open*.

| | | | | |
|---|---|---|---|---|
| 4 | 1.06 | 0.92 | 1.21 | 0.42 |
| 5 - Highest SES | 0.85 | 0.74 | 0.98 | 0.03 |
| Unknown | 1.08 | 0.89 | 1.32 | 0.44 |
| Liver history (ref=None) | | | | |
| Hepatitis B only | 0.96 | 0.74 | 1.26 | 0.78 |
| Hepatitis B and C | 0.92 | 0.71 | 1.19 | 0.54 |
| Hepatitis C only | 0.84 | 0.75 | 0.95 | 0.002 |
| Hepatitis C and heavy alcohol use | 0.70 | 0.61 | 0.80 | <.0001 |
| Heavy alcohol use | 1.01 | 0.85 | 1.21 | 0.89 |
| Heavy alcohol use and obesity | 0.69 | 0.55 | 0.86 | 0.001 |
| Obesity only | 1.03 | 0.86 | 1.24 | 0.77 |
| First Line therapy year (ref=2011) | | | | |
| 2012 | 0.95 | 0.75 | 1.20 | 0.65 |
| 2013 | 0.91 | 0.72 | 1.16 | 0.45 |
| 2014 | 0.94 | 0.75 | 1.19 | 0.63 |
| 2015 | 1.09 | 0.87 | 1.35 | 0.47 |
| 2016 | 0.99 | 0.78 | 1.26 | 0.95 |
| 2017 | 0.96 | 0.79 | 1.17 | 0.67 |
| 2018 | 1.00 | 0.80 | 1.25 | 0.98 |
| 2019 | 0.88 | 0.67 | 1.15 | 0.34 |
| 2020 | 0.95 | 0.69 | 1.31 | 0.77 |
| 2021 | 0.92 | 0.67 | 1.27 | 0.63 |
| 2022 | 0.87 | 0.64 | 1.19 | 0.39 |
| 2023 | 0.97 | 0.62 | 1.51 | 0.89 |
| ALBI grade (ref=1) | | | | |
| 2 | 1.69 | 1.54 | 1.86 | <.0001 |
| 3 | 2.65 | 2.22 | 3.16 | <.0001 |
| AFP (ref= <400) | | | | |
| >=400 | 0.99 | 0.92 | 1.06 | 0.69 |
| History of ascites (ref=No) | 1.76 | 1.58 | 1.96 | <.0001 |
| History of encephalopathy (ref=No) | 1.15 | 0.95 | 1.38 | 0.15 |
| History of varices (ref=No) | 0.91 | 0.74 | 1.13 | 0.39 |

HR = Hazard Ratio; CI = Confidence Interval
Patients with missing AFP and ALBI grade were excluded from these analyses.

© 2025 Lau-Min KS et al. *JAMA Network Open*.

**eTable 9.** Multivariable Cox proportional hazards regression model outputs evaluating the association between first-line systemic therapy type and progression-free survival (n=2716)

| | HR | 95% CI | | p-value |
|---|---|---|---|---|
| | | Lower limit | Upper limit | |
| First-line therapy (ref=Sorafenib) | | | | |
| Atezolizumab/bevacizumab | 0.84 | 0.70 | 0.99 | 0.04 |
| Durvalumab/tremelimumab | 0.82 | 0.53 | 1.26 | 0.36 |
| Lenvatinib | 0.91 | 0.76 | 1.09 | 0.32 |
| Nivolumab | 0.82 | 0.66 | 1.01 | 0.07 |
| Pembrolizumab | 0.87 | 0.60 | 1.27 | 0.47 |
| Age (ref=<50) | | | | |
| 50-64 | 0.82 | 0.66 | 0.997 | 0.046 |
| 65-74 | 0.75 | 0.59 | 0.95 | 0.02 |
| >=75 | 0.82 | 0.64 | 1.03 | 0.09 |
| Female (ref=Male) | 0.93 | 0.84 | 1.03 | 0.14 |
| Race (ref=White) | | | | |
| Asian | 0.92 | 0.76 | 1.10 | 0.36 |
| Black | 1.03 | 0.89 | 1.19 | 0.72 |
| Other race/unknown | 0.86 | 0.74 | 1.00 | 0.049 |
| Ethnicity (ref=Not Hispanic) | | | | |
| Hispanic | 0.99 | 0.83 | 1.19 | 0.95 |
| Unknown | 1.27 | 1.12 | 1.44 | 0.002 |
| Charlson comorbidity index (ref=0) | | | | |
| 1 | 1.13 | 1.03 | 1.23 | 0.01 |
| 2+ | 1.15 | 1.01 | 1.29 | 0.03 |
| Unknown | 1.00 | 0.72 | 1.40 | 0.99 |
| Insurance (ref=Commercial) | | | | |
| Dual Medicare and Medicaid | 0.84 | 0.70 | 1.02 | 0.07 |
| Medicaid | 1.05 | 0.88 | 1.24 | 0.62 |
| Traditional Medicare | 0.92 | 0.82 | 1.02 | 0.11 |
| Medicare Advantage | 0.88 | 0.78 | 1.00 | 0.045 |
| Other | 1.01 | 0.90 | 1.15 | 0.84 |
| SES (ref=1-Lowest) | | | | |
| 2 | 1.07 | 0.89 | 1.28 | 0.48 |
| 3 | 1.11 | 0.98 | 1.25 | 0.11 |

© 2025 Lau-Min KS et al. *JAMA Network Open*.

| | | | | |
|---|---|---|---|---|
| 4 | 1.14 | 1.01 | 1.28 | 0.04 |
| 5 - Highest SES | 1.02 | 0.88 | 1.19 | 0.77 |
| Unknown | 1.04 | 0.87 | 1.23 | 0.70 |
| Liver history (ref=None) | | | | |
| Hepatitis B only | 1.00 | 0.80 | 1.26 | 0.99 |
| Hepatitis B and C | 1.05 | 0.81 | 1.36 | 0.70 |
| Hepatitis C only | 0.86 | 0.76 | 0.97 | 0.01 |
| Hepatitis C and heavy alcohol use | 0.71 | 0.62 | 0.82 | <.0001 |
| Heavy alcohol use | 0.99 | 0.86 | 1.13 | 0.85 |
| Heavy alcohol use and obesity | 0.84 | 0.70 | 1.00 | 0.06 |
| Obesity only | 1.16 | 0.97 | 1.38 | 0.11 |
| First line therapy year (ref=2011) | | | | |
| 2012 | 1.11 | 0.84 | 1.47 | 0.45 |
| 2013 | 1.04 | 0.81 | 1.33 | 0.77 |
| 2014 | 1.13 | 0.84 | 1.53 | 0.42 |
| 2015 | 1.14 | 0.85 | 1.54 | 0.38 |
| 2016 | 1.27 | 0.93 | 1.72 | 0.13 |
| 2017 | 1.15 | 0.92 | 1.44 | 0.22 |
| 2018 | 1.21 | 0.92 | 1.60 | 0.17 |
| 2019 | 1.21 | 0.89 | 1.63 | 0.22 |
| 2020 | 1.19 | 0.93 | 1.53 | 0.17 |
| 2021 | 1.18 | 0.88 | 1.58 | 0.28 |
| 2022 | 1.07 | 0.80 | 1.43 | 0.66 |
| 2023 | 1.40 | 1.06 | 1.86 | 0.02 |
| ALBI grade (ref=1) | | | | |
| 2 | 1.33 | 1.21 | 1.45 | <.0001 |
| 3 | 1.75 | 1.46 | 2.10 | <.0001 |
| AFP (ref= <400) | | | | |
| >=400 | 1.01 | 0.95 | 1.08 | 0.75 |
| History of ascites (ref=No) | 1.46 | 1.28 | 1.67 | <.0001 |
| History of encephalopathy (ref=No) | 1.01 | 0.83 | 1.24 | 0.91 |
| History of varices (ref=No) | 0.95 | 0.73 | 1.24 | 0.70 |

HR = Hazard Ratio; CI = Confidence Interval

Patients with missing AFP and ALBI grade were excluded from these analyses

© 2025 Lau-Min KS et al. *JAMA Network Open*.

**eFigure 1.** Patterns in second-line systemic therapy administration, by first-line therapy type

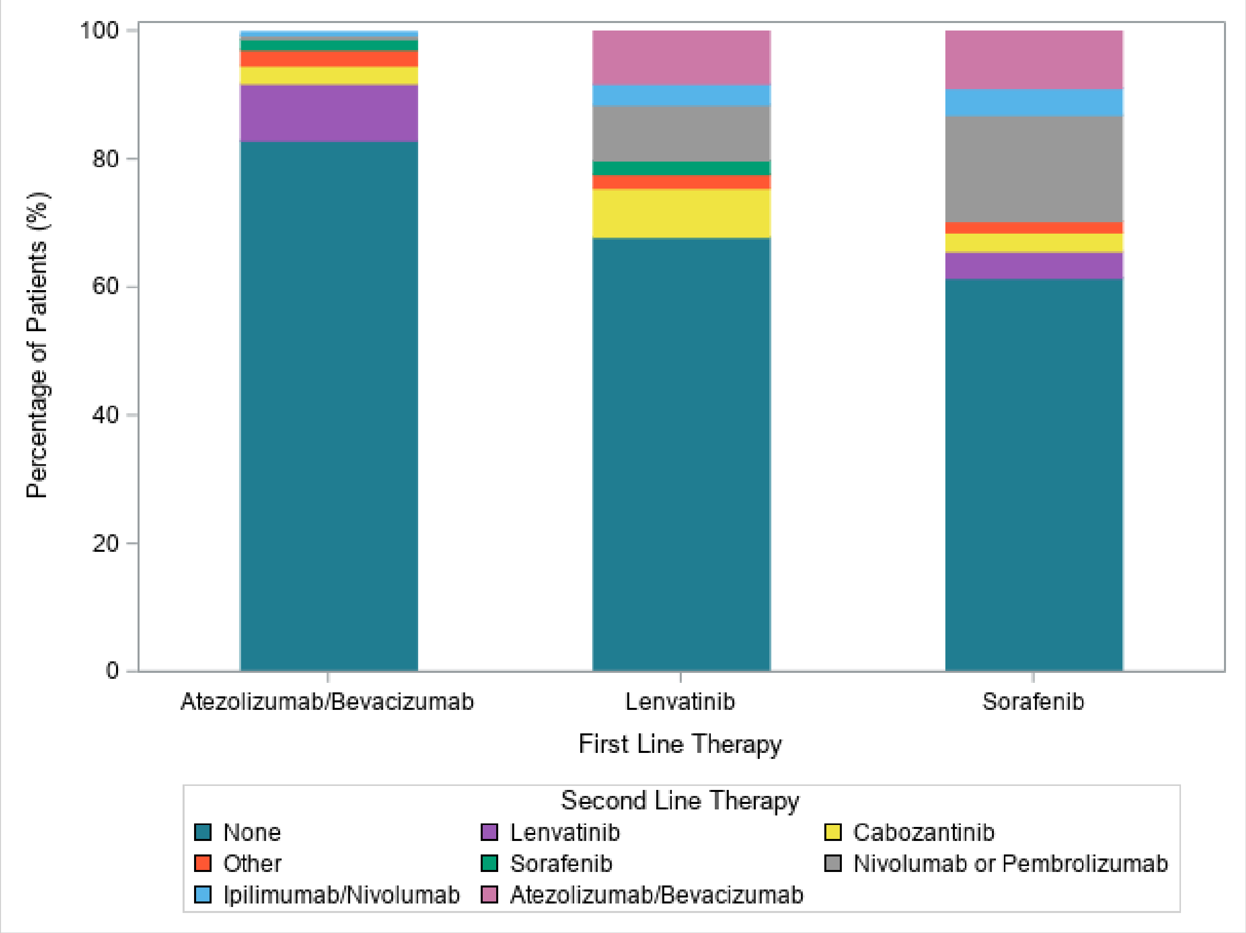


© 2025 Lau-Min KS et al. *JAMA Network Open*.

**eFigure 2.** Percentage of patients who received locoregional therapy after initiating systemic therapy for hepatocellular carcinoma.

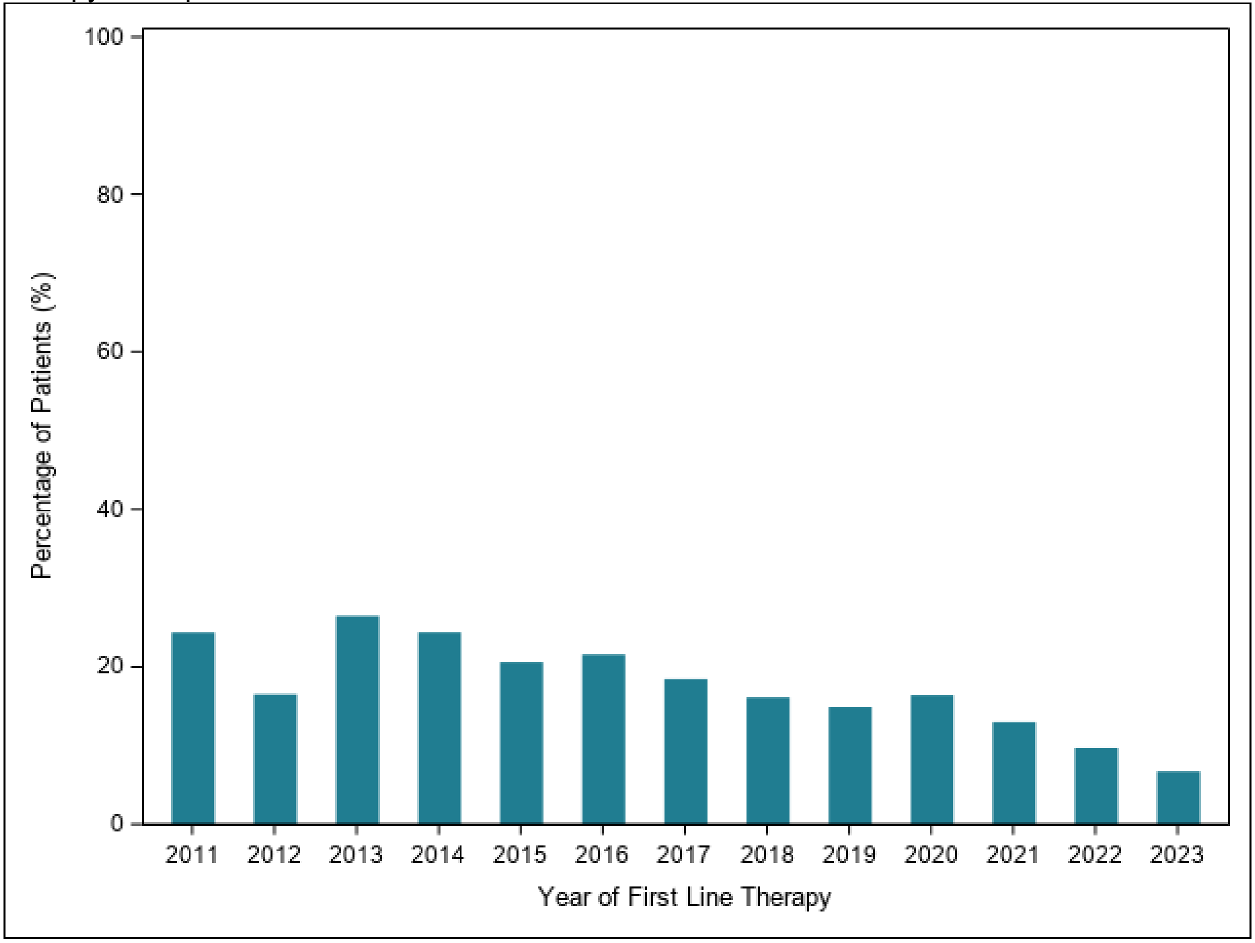


© 2025 Lau-Min KS et al. *JAMA Network Open*.
