# Supplementary material for: Treatment Patterns and Survival Outcomes Among Patients With Hepatocellular Carcinoma
Source: JAMA Netw Open. 2025 Dec 30;8(12):e2551665. doi: 10.1001/jamanetworkopen.2025.51665 (PMC12754679; doi:10.1001/jamanetworkopen.2025.51665)
Supplement: Supplement 2. — Data Sharing Statement [file jamanetwopen-e2551665-s002.pdf]

Data Sharing Statement

Lau-Min. Treatment Patterns and Survival Outcomes Among Patients With Hepatocellular Carcinoma. *JAMA Netw Open*. Published December 30, 2025.
doi:10.1001/jamanetworkopen.2025.51665

Data
Data available: Yes
Data types: Deidentified participant data, Data dictionary
How to access data: The study data were originated by Flatiron Health, Inc. These de-identified data may be made available upon request subject to a license agreement with Flatiron Health (DataAccess@flatiron.com).
When available: With publication

Supporting Documents
Document types: None

Additional Information
Who can access the data: Researchers whose proposed use of the data has been approved
Types of analyses: Any purpose
Mechanisms of data availability: After approval of a proposal and with a signed data access agreement
